# Supplementary material for: Integrating ensemble machine learning and multi-omics approaches to identify Dp44mT as a novel anti-Candida albicans agent targeting cellular iron homeostasis
Source: Front Pharmacol. 2025 Apr 24;16:1574990. doi: 10.3389/fphar.2025.1574990 (PMC12058677; doi:10.3389/fphar.2025.1574990)
Supplement: Supplementary file 2 [file DataSheet1.docx]

Supplementary Material 1

# Supplementary Figures
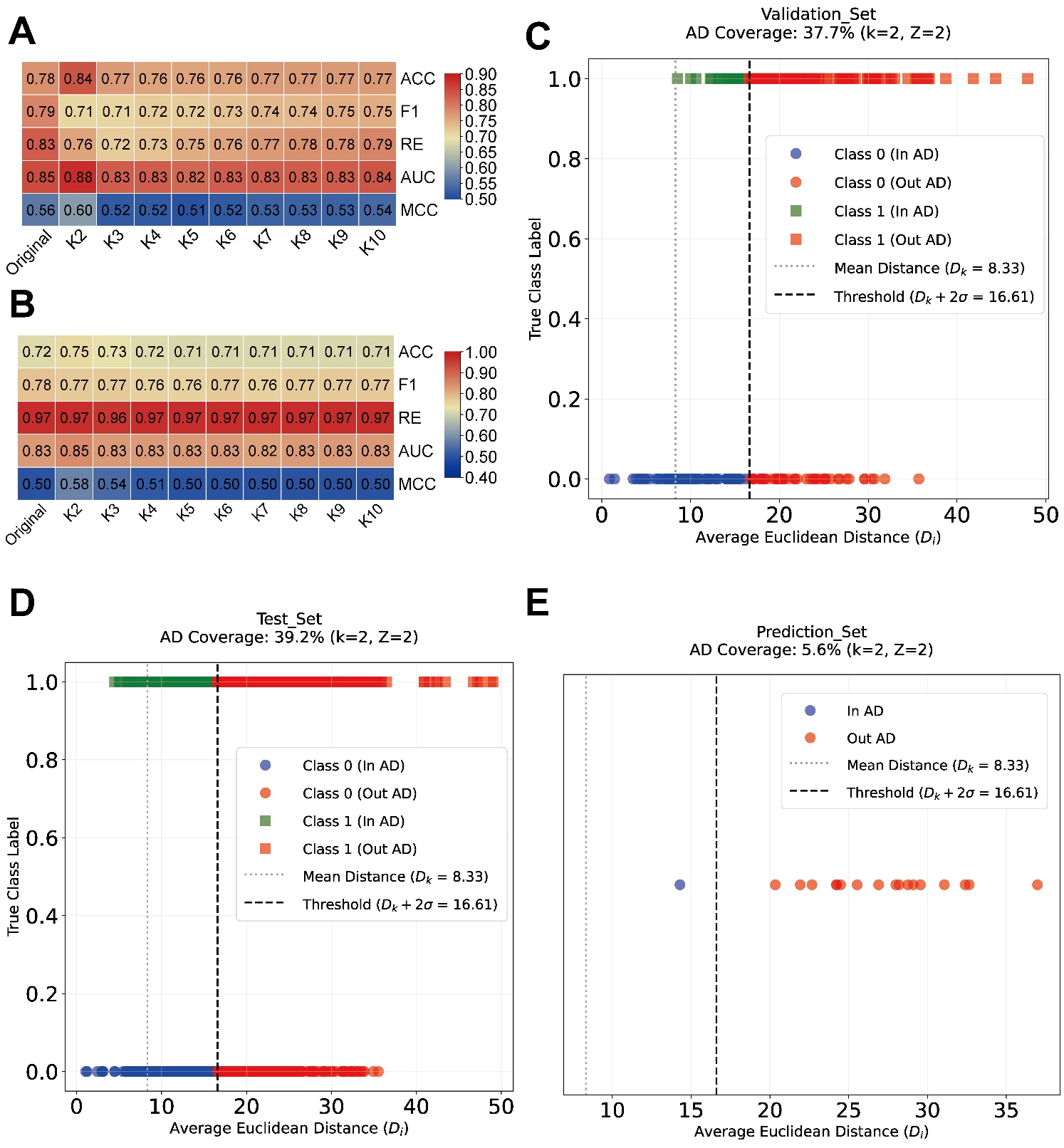


**Supplementary Figure 1.** Applicability domain (AD) analysis. Performance analysis of the ensemble model in the AD-only validation set 1 **(A)** and validation set 2 **(B)** across varying k-values. The AD coverage varied among the datasets, comprising **(C)** validation set 1, **(D)** validation set 2, and **(E)** the compounds in-house library predicted to be active through ensemble learning. Dₖ represents the mean distance while σ indicates the standard deviation among the K-Nearest Neighbors Euclidean distances in the training set.


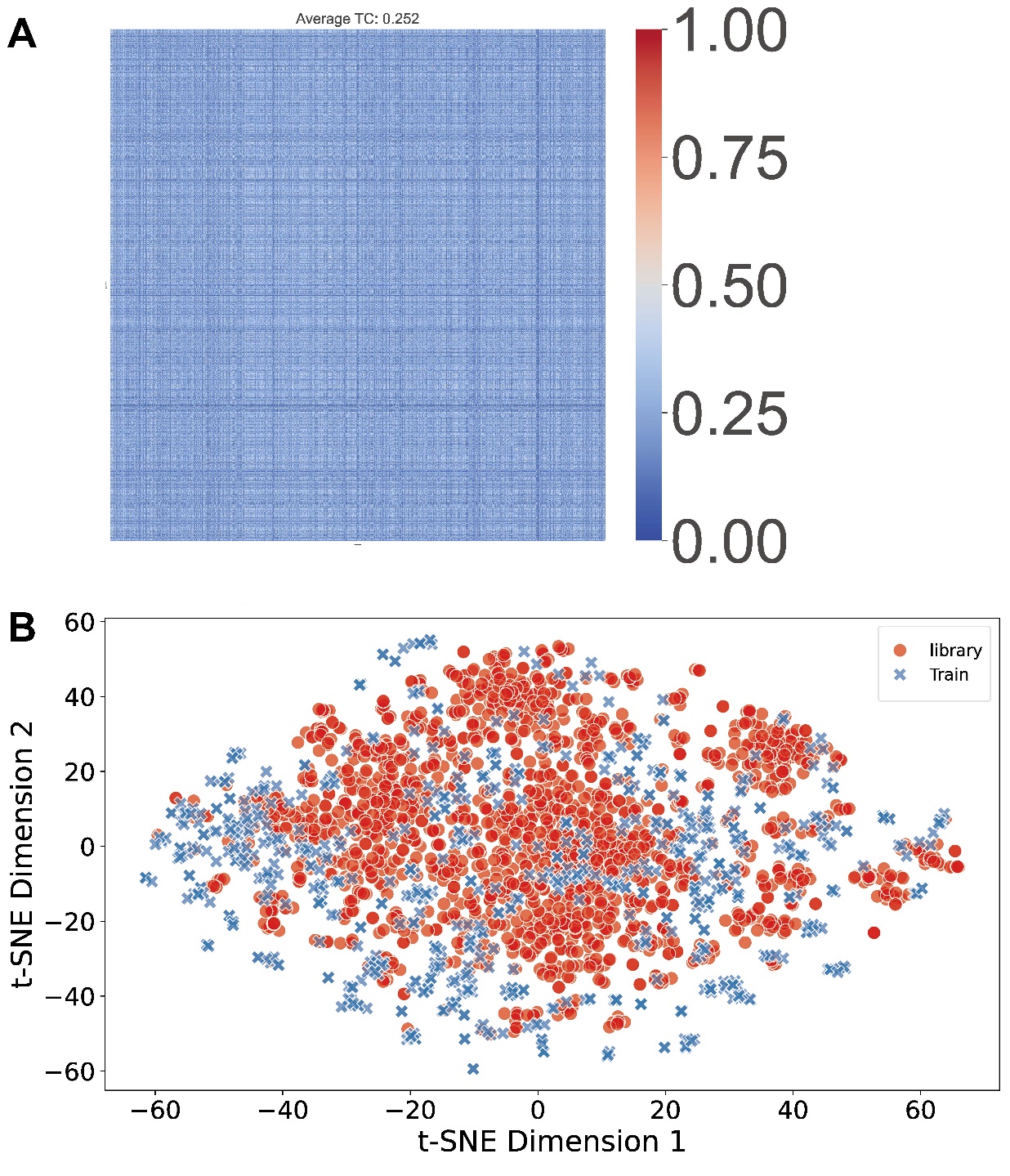


**Supplementary Figure 2.** **(A)** The Tanimoto coefficient (TC) heatmap of in-house library compounds versus training set compounds. **(B)** The chemical space distribution of compounds in the library versus those in the training set.


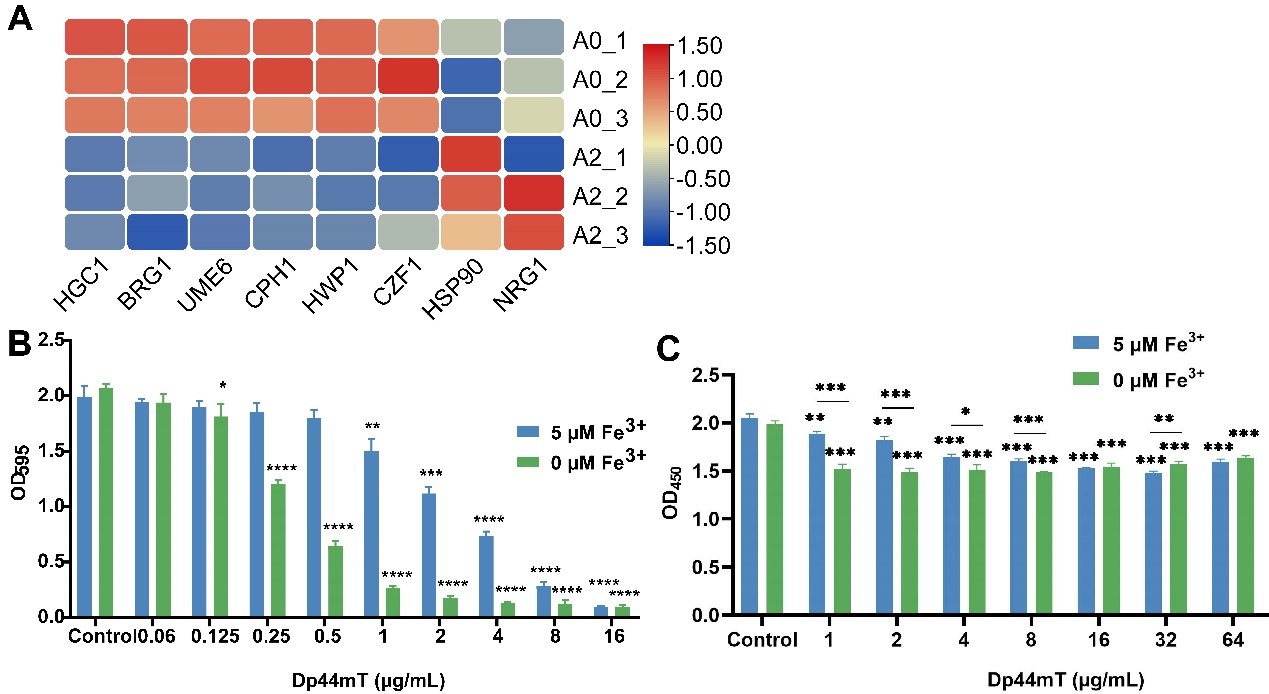


**Supplementary Figure 3.** Anti-hypha and -biofilm property of Dp44mT. **(A)** Expression of hyphal-associated genes after treatment with 2 µg/mL of Dp44mT for 6 hours. **(B)** Impact of Dp44mT on biofilm formation assessed by crystal violet test. **(C)** XTT assay to detect the extent to which Dp44mT disrupt mature biofilms. Statistical significance between the two groups was assessed using a t-test. ‘*’ indicates ‘p < 0.05’; ‘**’ indicates ‘0.001 < p ≤ 0.01’; ‘***’ indicates ‘0.0001 < p ≤ 0.001’; ‘****’ indicates ‘p ≤ 0.0001’.


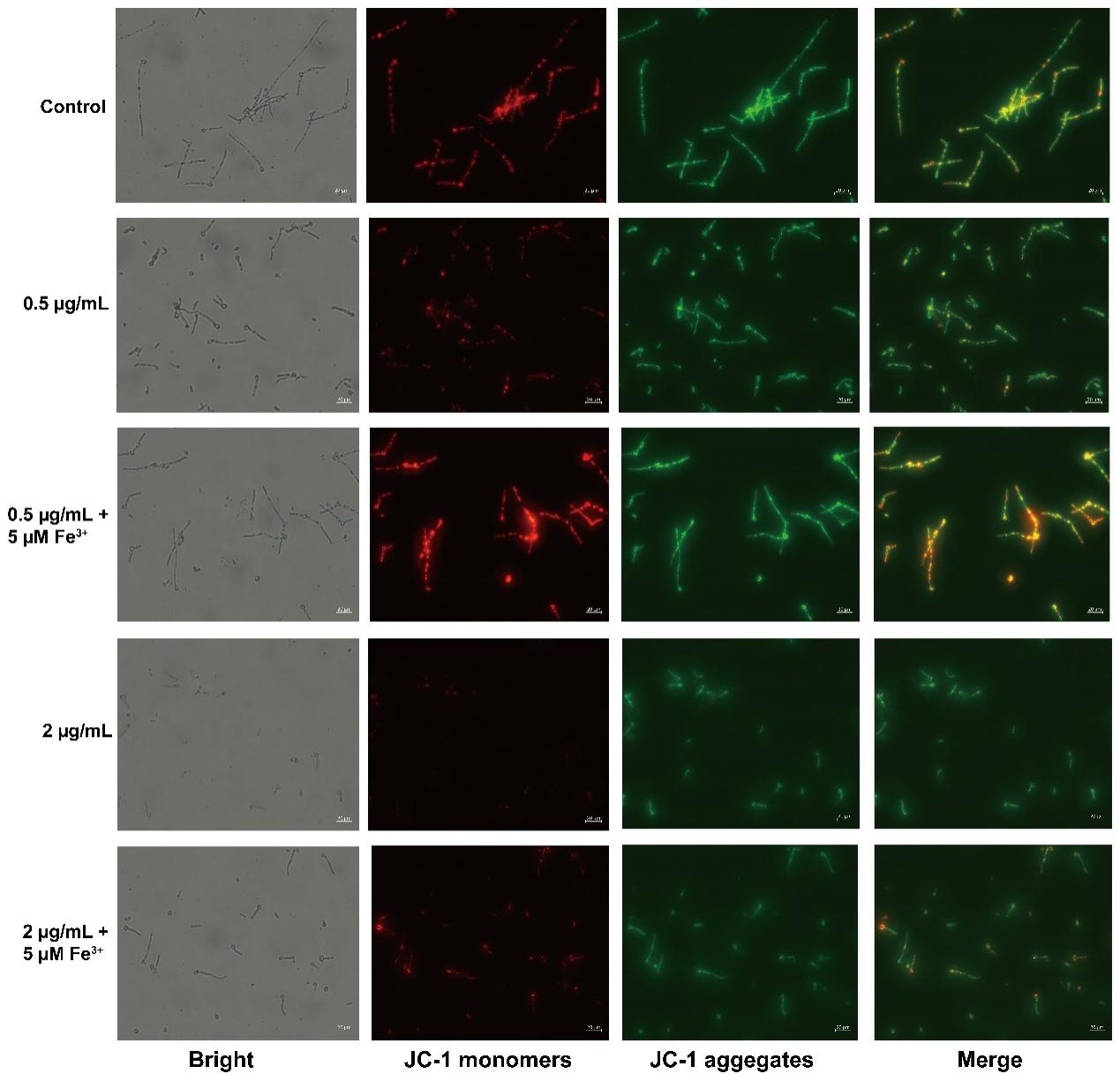


**Supplementary Figure 4.** Observation of C. albicans cells stained with JC-1 for MMP. C. albicans cells were incubated for 10 hours with Dp44mT at various concentrations in RPMI 1640 medium with or without 5 µM Fe^3+^, followed by staining with JC-1.


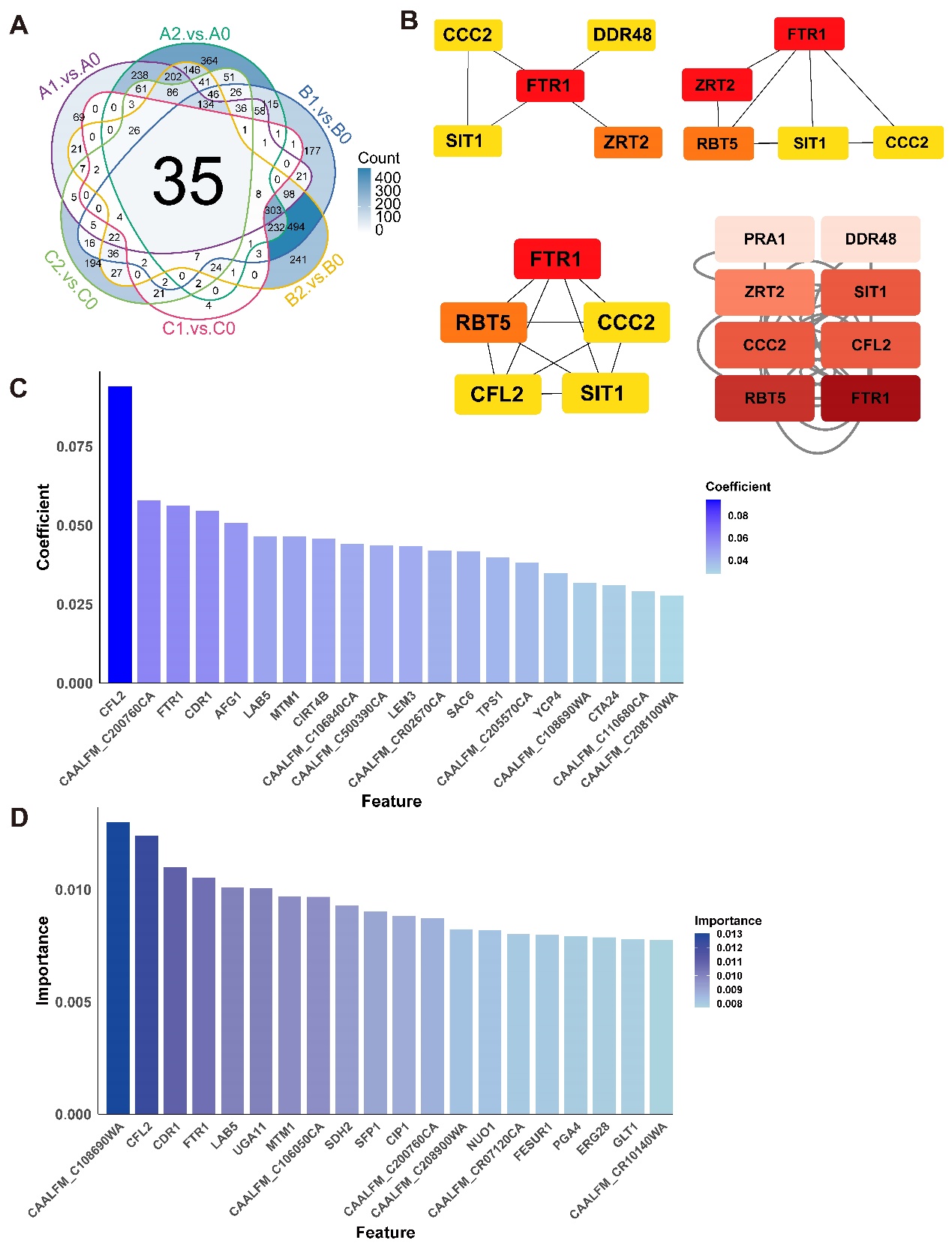


**Supplementary Figure 5.** Identifying the hub genes. **(A)**The overlapping proteins between the transcriptomics and proteomics. **(B)** The algorithm of BottleNeck, Stress, EPC, MCC, MNC, and Radiality from the cytoHubba plugin and the algorithm of Betweenness, Degree, and Closeness from cytoNCA plugin for identified hub genes. The top 20 hub genes identified by SVM-RFE **(C)** and Logistic Regression **(D)**, ranked by coefficient and importance.


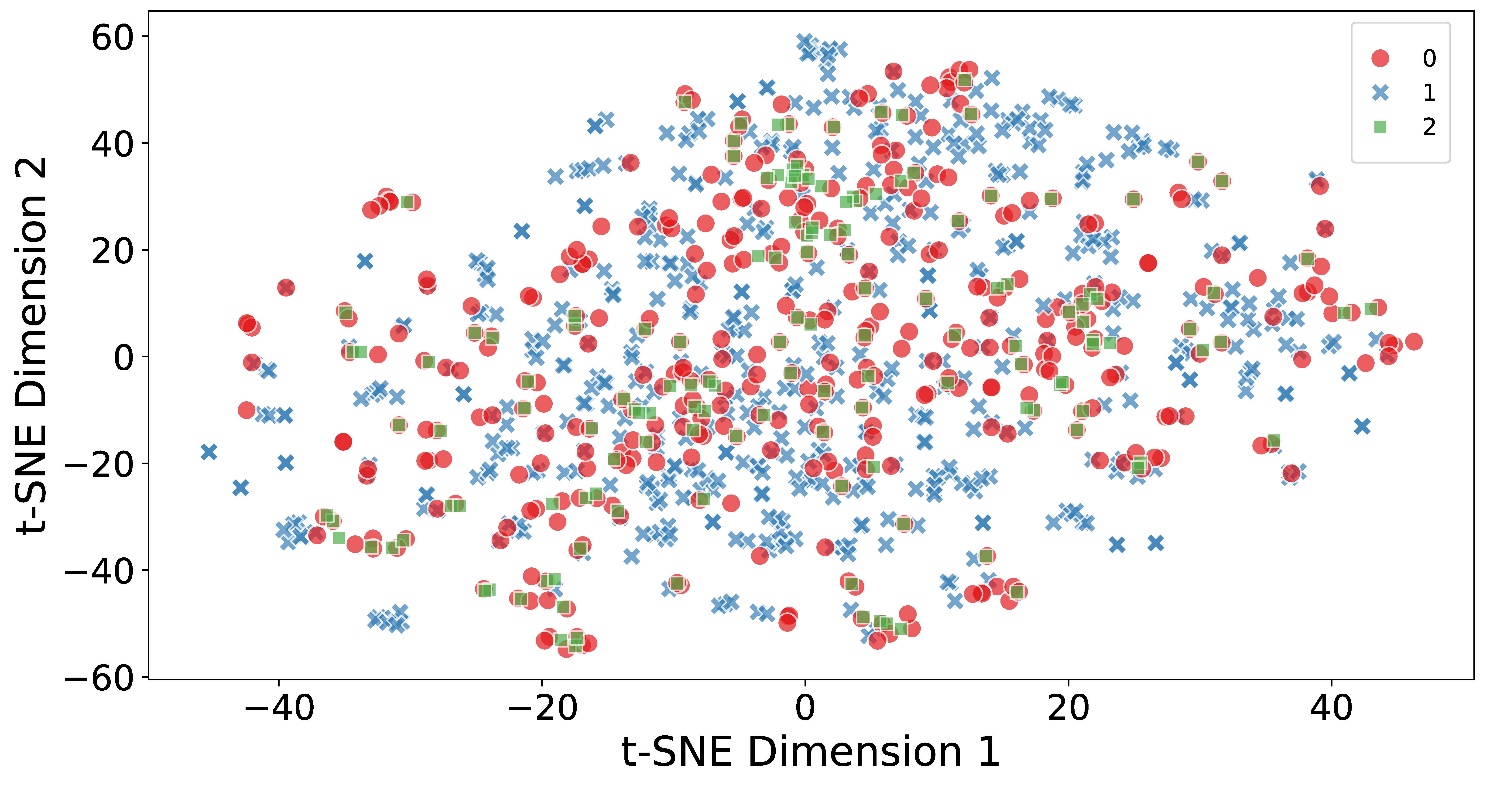


**Supplementary Figure 6.** Structural diversity of compounds across different types. 0 denotes inactive compounds, 1 represents active compounds, and 2 indicates SMOTE-synthesized compound in the inactive group.


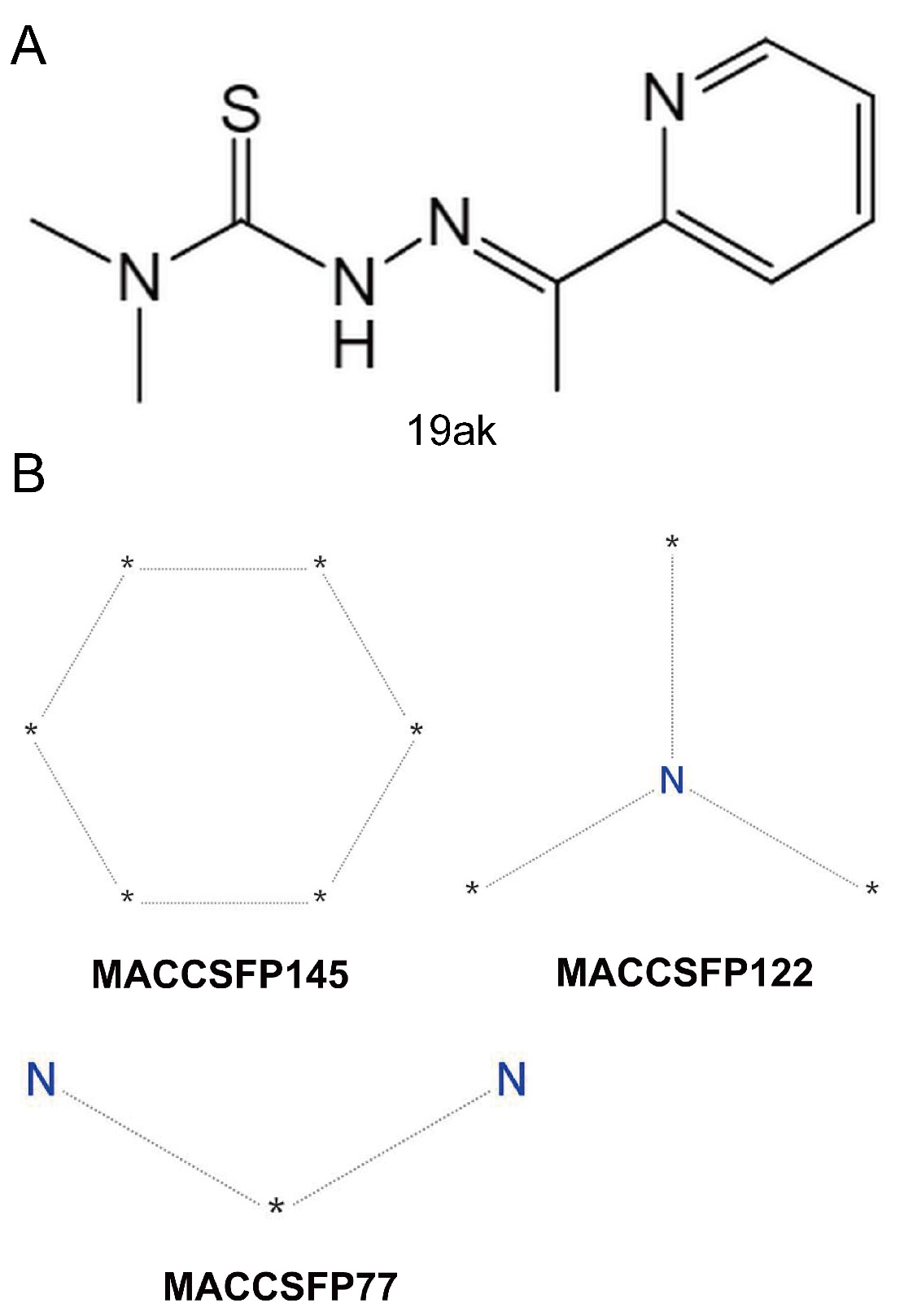


**Supplementary Figure 7.** The structure of 19ak (A) and it’s core scaffold (B).
